# Describing Dietary Habits and Body Composition Among High-Intensity Functional Training Athletes: A Mixed Methods Approach

**DOI:** 10.3390/sports13100340

**Published:** 2025-10-02

**Authors:** Kworweinski Lafontant, Jack Livingston, Sofea Smith, Michelle A. Da Silva Barbera, Claudia Gonzalez, Susan Kampiyil, Ngoc Linh Nhi Nguyen, Blake Johnson, Jeffrey R. Stout, David H. Fukuda

**Affiliations:** 1 Physiology of Work and Exercise Response (POWER) Lab, Institute of Exercise Physiology and Rehabilitation Science, University of Central Florida, Orlando, FL 32816, USA; kworweinski.lafontant@ucf.edu (K.L.); sofea.smith@ucf.edu (S.S.); mi890893@ucf.edu (M.A.D.S.B.); claudiagonzalezabadin@gmail.com (C.G.); li335407@ucf.edu (N.L.N.N.); jeffrey.stout@ucf.edu (J.R.S.); 2 ApexFit Lake Nona, Orlando, FL 32832, USA

**Keywords:** nutrition guidelines, supplementation, recreational athletes, fitness, bioimpedance, BodPod

## Abstract

High-intensity functional training (HIFT) has grown in popularity in the past several decades, yet previous research has largely focused on the dietary habits and body composition of elite HIFT athletes and utilized only quantitative study designs, potentially limiting our understanding of typical HIFT athletes. This study aimed to comprehensively describe the common dietary habits and body composition of HIFT athletes. Data were only analyzed descriptively. Among 62 HIFT athletes (age: 36 ± 11.7 years), we estimated body fat percentage (BF%) using a Siri 3-compartment model, and we assessed dietary habits, dietary supplement (DS) use, and open-response rationales for DS use/disuse via an online questionnaire. Qualitative data from open-response questions were coded and grouped via inductive thematic analysis. Body composition varied among both male (n = 36, BF% = 6.5–27.6%) and female participants (n = 26, BF% = 10.6–37.6%). Most participants reported regular consumption of lean meats and home-cooked meals, yet few participants (~20%) regularly consumed the recommended twice daily servings of dairy, fruits, vegetables, and whole grains. Most (77.4%) HIFT athletes reported DS use, with the average HIFT athlete using approximately six DS; dairy protein, creatine, caffeine, and electrolyte drinks were the most reported DS. Improving health, recovery, and nutrient intake were common reasons for using DS, whereas a lack of noticeable results was the most common reason for discontinuation. Some HIFT athletes may rely on DS to address nutrient gaps rather than whole foods.

## 1. Introduction

High-intensity functional training (HIFT) is a unique type of training that has increased in popularity globally since its formal inception in the year 2000 by the CrossFit^®^ organization. CrossFit^®^’s own website estimates that five million people (who they term “athletes”) participate in CrossFit^®^ [1], with many others opting to participate in HIFT outside of the CrossFit^®^ organization (e.g., boot-camp style workouts, F45^®^, Hyrox^®^, etc.; Table 1). HIFT workouts vary in duration and intensity, with a blended focus on weightlifting, gymnastics, and metabolic conditioning [2]. These workouts are performed in solo or team settings, and CrossFit^®^ athletes can compete in local, regional, national, and international competitions as well, aiming to complete a given workout in the fastest time with the best performance. Beyond competitive athletics, HIFT also caters to the general population who may only seek general health and wellness. However, despite the global popularity of HIFT, research on this style of training has largely been limited to elite competitive athletes (e.g., CrossFit^®^ games competitors). This leaves a gap in our understanding of who the typical HIFT athlete is, specifically regarding their dietary habits and body composition.

Generally, the common nutritional practices and dietary supplements used by athletes are to improve the specific athletic performance demands of their sport. From this, researchers and clinicians can predict the common dietary supplements used by athletes, leading to further research and sports nutrition guidelines/recommendations. However, given the various athletic requirements for HIFT performance (e.g., muscular endurance, aerobic endurance, muscular strength, etc.), a multitude of different ergogenic effects may be beneficial, making it difficult to determine which dietary strategies are most common. Much of the previous research on this topic has focused on the efficacy of different investigator-initiated dietary interventions in improving HIFT performance [6], yet little is known about the typical dietary practices and supplementation of HIFT athletes. A systematic review and meta-analysis by Knapik et al. [7] included 108 unique studies detailing the prevalence of dietary supplement use among a variety of sports; none of those studies focused on HIFT athletes. In studies specific to HIFT (CrossFit^®^, in particular), Brisebois et al. [8], Gogojewicz et al. [9], and Pearson and Jenkins [10] have descriptively reported dietary habits among athletes. However, the characterization of participants in those studies was limited, as only Gogojewicz et al. [9] reported body composition alongside dietary habits. As these descriptive studies are used to establish the norm for HIFT athletes and may lead to future research, examining dietary habits in tandem with body composition may be critical given how dietary habits are well understood to influence body composition.

Previous work by Menargues-Ramírez et al. [11] examined body composition in 27 CrossFit^®^ athletes regarding their performance in standard workouts such as Fran and Kelly. Albeit with a small sample size, their results provide some insight into the typical body composition of CrossFit^®^ athletes and how body composition relates to performance within the sport. Likewise, de Sousa et al. [12] compared body mass and body fat percentage between 13 resistance trained men and 13 CrossFit^®^ trained men, reporting no significant differences, yet also not reporting any data on other facets of body composition or female athletes. Mangine et al. [2] provided greater detail on body composition among CrossFit^®^ athletes, with data on bone mineral content and muscle morphology. However, similar limitations persisted with a small sample of only 16 CrossFit^®^ athletes represented [2]. There is a paucity of studies that examine body composition among HIFT athletes with a relatively large sample size. Cebrián-Ponce et al. [13] included 145 Spanish CrossFit^®^ athletes and examined somatotypes and body composition. While their sample size was robust, their inclusion criteria limited participation only to highly trained CrossFit^®^ athletes [13], which may not be applicable to the wide variety of HIFT athletes, both competitive and non-competitive.

While some past works have examined body composition or dietary habits alone, the two domains are interconnected and should be examined together. Furthermore, the past research on HIFT athletes has largely been quantitative, despite the value in practitioners and coaches learning about the qualitative experiences of similar athletes. For practitioners and coaches, it is also critical to understand the body composition of HIFT athletes when appraising their common dietary habits to ensure that those dietary habits are applicable. Therefore, the primary purpose of this study was to comprehensively describe the common dietary habits and body composition of HIFT athletes.

## 2. Materials and Methods

### 2.1. Study Design

In line with our purpose, this study utilized a descriptive design to assess HIFT athletes, as no inferential or comparative variables were of interest. Using a mixed-methods cross-sectional study design, we assessed 62 HIFT athletes from the greater Orlando, FL, metropolitan area from October 2024 to March 2025. Participants were recruited via word-of-mouth, social media postings (e.g., Facebook groups, Twitter/X, Instagram, etc.), and fliers posted at local HIFT gyms. Adults above the age of 18 years with at least 6 months of HIFT experience were included in this study. Those with uncontrolled metabolic diseases (e.g., dyslipidemia, diabetes; self-reported via health history questionnaire), self-reported body modifications/implants of any kind, missing limbs, self-reported pregnancy, or current medication use that may confound body composition assessments (e.g., antidiuretics; self-reported via health history questionnaire) were excluded from participation in this study. All study procedures were approved by the University of Central Florida Institutional Review Board (ID: STUDY00007054) and carried out in accordance with the Declaration of Helsinki. All participants provided written informed consent prior to participation.

**Figure 1:**
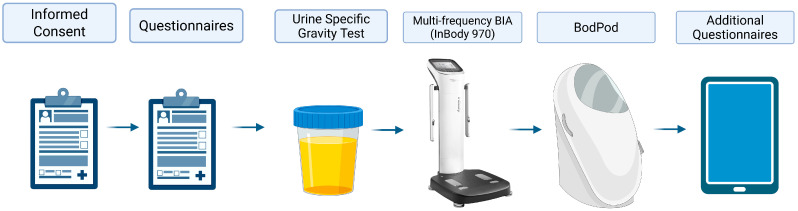
Flow of study procedures Reproduced or adapted from [14], with permission from *MDPI*, 2025.

Figure 1 provides a simplified overview of study procedures. All assessments were completed in a climate-controlled environment monitored via digital weather station (FUNcaster Barometer, TecScan, Gainesville, FL, USA; temperature = 22.1 ± 1.2 °C, humidity = 52.4 ± 8.1%, barometric pressure = 770.0 ± 61.3 mmHg), and the order of assessments was kept the same for all participants. After providing informed consent, participants self-reported sex, age, and race/ethnicity, which a trained research assistant recorded on a data collection form. Participants were then given a paper health history questionnaire to assess health status and confirm eligibility for participation in the study.

### 2.2. Rapid Eating Assessment for Participants

We utilized the shortened Rapid Eating Assessment for Participants (REAP-S, version 1) as part of our assessment of dietary habits. The REAP-S is a standardized assessment of dietary habits, designed for assessing food intake habits through 13 Likert-style questions [15]. Previous research has validated the REAP-S against other standard food frequency questionnaires, showing significant correlations between nutrient intake and serving size intake from food frequency questionnaires and the REAP-S, respectively [15]. Participants completed the REAP-S via paper while supervised, and responses were included in our analysis as-is, without scoring.

### 2.3. Bioelectrical Impedance Analysis

We utilized a phase-sensitive, direct segmental multifrequency InBody 970^®^ (InBody BWA, Audubon, PA, USA) device for BIA assessments. The InBody 970^®^ performed self-calibration each morning prior to assessments. Participants were instructed to arrive at the study visit having fasted (i.e., no food or beverages) for at least 3 h, avoided caffeine for at least 12 h, and abstained from alcohol and strenuous physical activity/exercise for at least 24 h prior. Additionally, participants were instructed to wear minimal, light, compression/skin-tight athletic clothing. We collected a mid-stream urine sample from each participant to assess hydration status via urine specific gravity (USG) using a MISCO PA202x Palm Abbe digital refractometer (MISCO Refractometer, Solon, OH, USA). Participants had to have a USG of <1.030 to be considered adequately hydrated; those with USG values ≥ 1.030 were rescheduled to complete body composition testing on another day with sufficient hydration.

After ensuring adequate hydration and that pre-visit protocols were met (via self-report), we assessed height in centimeters (cm) using a stadiometer (Health-O-Meter^TM^, Model 402KL, McCook, IL, USA). Participants were then given an InBody^®^ Tissue (InBody BWA, Audubon, PA, USA) and were instructed to wipe the palms of their hands and bottom of their feet, then step onto the InBody 970^®^ to follow all on-screen instructions.

From the InBody 970^®^, we extracted whole-body values for phase angle (PhA), impedance (Z), resistance (R), and reactance (Xc) at 50 kHz, as well as Z at 250 and 5 kHz for the calculation of impedance ratio (IR) (Z_250kHz_/Z_5kHz_). These bioelectrical variables are used as indicators of global cellular health, with background information provided elsewhere [16,17]. Body mass, body fat mass, fat-free mass, body fat percentage, skeletal muscle mass, and total body water values were also extracted directly from the InBody 970^®^ and are reported in Appendix A. Total body water from the InBody 970^®^ was used to estimate body composition within the Siri 3C model [18].

### 2.4. Air Displacement Plethysmography

Following the BIA assessment, participants completed a BodPod^®^ (version 5.4.6, DLL version 3.90, controller version 13.60, COSMED, Rome, Italy) air displacement plethysmography assessment. Immediately prior to each study visit, the warm-up, scale, volume, hardware, and autorun calibrations were completed on the BodPod^®^ according to device specifications. Following calibrations, non-bald participants were given a nylon compressive swim cap to cover head hair, and body mass was assessed using the BodPod^®^’s scale. Participants were then instructed to sit in the BodPod^®^ silently, motionless, and with normal breathing for the duration of the test. Each test was comprised of two-to-three consecutive trials of assessments as determined by the BodPod^®^, with each trial lasting approximately 2 min. Thoracic gas volume was estimated for each assessment using the BodPod^®^’s built-in equation.

From the BodPod^®^, body fat percentage, fat mass, fat-free mass, body mass, and body density were extracted. We utilized total body density and body mass from the BodPod^®^ to estimate body fat percentage, fat mass, and fat-free mass estimates via Siri’s 3-compartment model [18], detailed in Equations (1)–(3) below (with units provided in brackets), as our primary estimates of body composition.

Body Fat Percentage [%] = 2.118/Density [kg/L] − 0.78 * (Total Body Water [L]/Body Mass [kg]) − 1.354(1)

Fat Mass [kg] = Body Mass [kg] * Body Fat Percentage [%](2)

Fat-Free Mass [kg] = Body Mass [kg] − Fat Mass [kg](3)

### 2.5. Questionnaires

After completing all body composition assessments, participants were given several questionnaires to complete on an iPad via Qualtrics software (https://www.qualtrics.com/) (Qualtrics, Provo, UT, USA) while supervised. These questionnaires included the 9-item short version of the International Physical Activity Questionnaire (IPAQ), which is a standardized questionnaire used to quantify physical activity levels. We also included a survey regarding HIFT participation, asking participants to self-report the duration, frequency, and competitive status of their participation in HIFT. Participants also completed a survey regarding dietary supplements and performance enhancing drugs if they used those aids, respectively. This survey asked participants to self-report their use, disuse, rationale(s) for use/disuse, and beliefs of the most beneficial dietary supplements and performance enhancing drugs for HIFT athletes. The dietary supplement and performance enhancing drug surveys both included multiple choice and free response open-ended questions.

### 2.6. Statistical Analysis

Data were stored and organized within Microsoft Excel (Version 2505, Microsoft Corporation, Redmond, WA, USA) and then analyzed descriptively with jamovi version 2.5.6 [19]. Qualitative data from the questionnaires’ open-ended responses was analyzed independently by KL using inductive thematic analysis. Participant responses were reviewed to develop initial codes, which were then further grouped into final themes. These themes were reviewed by the co-authors for consensus regarding their clarity. The purpose of this study was descriptive, so no further statistical analyses were conducted. All quantitative data are reported as mean ± standard deviation and ranges (i.e., minimum and maximum values) unless indicated otherwise.

## 3. Results

### 3.1. Demographics and HIFT Participation

Table 2 lists demographic information for the participants. Participants ranged in age from 18 to 64 years. Most participants were recreational HIFT athletes (n = 39, 62.9%), while 37.1% (n = 23) were competitive. Of the competitive HIFT athletes, 100% (n = 39) competed locally, 11.3% (n = 7) competed regionally, and 6.5% (n = 4) competed nationally.

**Figure 2:**
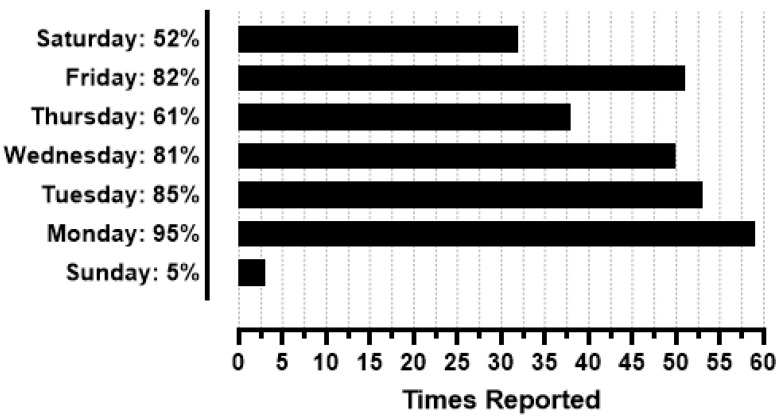
Days of the week in which participants complete HIFT workouts (N = 62).

Figure 2 illustrates the days when participants regularly complete HIFT workouts. Participants reported completing an average of 4.9 ± 2.8 HIFT workouts per week. The morning time (4:00 am–12:00 pm) was the most popular time for HIFT workouts (n = 37, 59.7%), with participants also reporting the midday (12:00 pm–4:00 pm; n = 14, 22.6%), evening (4:00 pm–9:00 pm; n = 19, 30.6%), and late night (9:00 pm–4:00 am; n = 4, 6.5%) as times that they complete HIFT workouts.

### 3.2. Body Composition

Table 3 provides the body composition of participants based on the Siri 3C model. Body composition data directly exported from the InBody 970^®^ are provided in Appendix A.

### 3.3. Dietary Habits

Table 4 lists the common dietary habits of participants based on the REAP-S survey. Most participants prefer to cook meals at home rather than ordering food from restaurants (n = 61, 98.4%). Most participants also avoid skipping breakfast, ultra-processed meats, fried foods, regular snack chips, discretionary fats (e.g., butter, margarine), and sugar-sweetened beverages. No clear majority of participants regularly achieve 2 servings per day of whole grains, fruits, vegetables, or dairy products.

**Figure 3:**
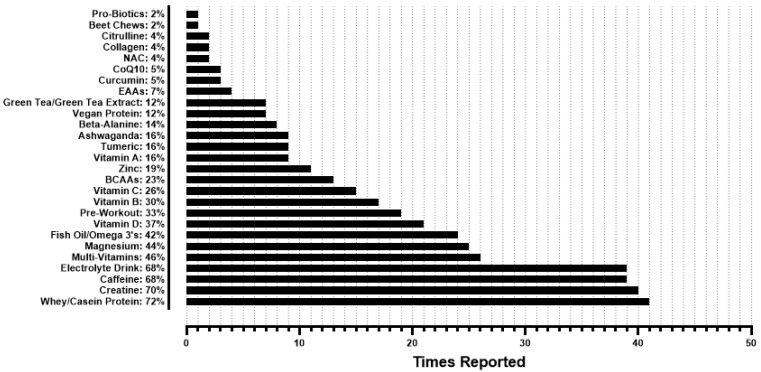
Frequencies of dietary supplements reported by participants that use supplements (n = 57). BCAAs = branched chain amino acids; EAAs essential amino acids; CoQ10 = coenzyme Q10; NAC = N-acetylcysteine.

Only one (1.6%) participant reported using performance-enhancing drugs, so data from those questions are not reported herein. Regarding dietary supplements, 48 (77.4%) participants used dietary supplements at the time of data collection, with 9 (14.5%) reporting past use of dietary supplements that have since been discontinued, and 5 (8.1%) that never used dietary supplements. Of the participants that have used dietary supplements (n = 57), an average of 6.4 ± 4.4 different supplements were used per athlete, ranging from 0 to 16. Figure 3 provides the frequencies of dietary supplements being reported by participants. Whey/casein protein, creatine, caffeine, electrolyte drinks, and multi-vitamins were the top five reported dietary supplements by participants.

**Figure 4:**
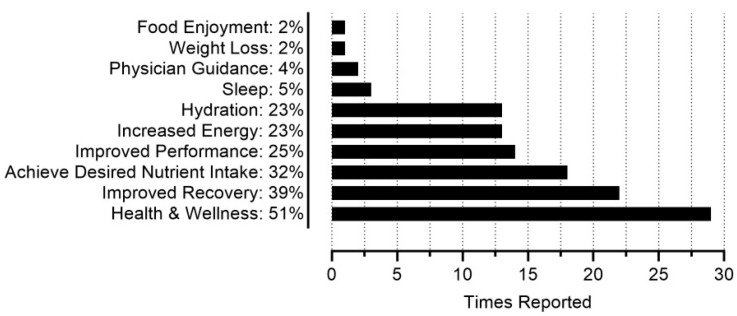
Frequencies for dietary supplement use rationale (n = 57).

Figure 4 provides the frequencies of final theme categorization from participants’ open responses regarding their rationale for dietary supplement use (n = 57). Health & wellness, improved recovery, achieving a desired nutrient intake, improved performance, and increased energy were the most common reasons for dietary supplement use among participants. For health & wellness, participants reported using “magnesium and zinc as a general health supplement,” “fish oil for brain and heart health,” and “collagen to support soft tissue,” among other examples. For improved recovery, participants reported using “magnesium for muscle recovery,” “creatine to help build and restore muscles,” and “protein for decreased recovery time,” among other examples. For achieving a desired nutrient intake, participants mention using “whey protein to help hit protein targets,” “vitamins to supplement the diet/make up for any micronutrients I might be missing,” and “vitamin D to supplement daily vitamin D recommendations,” among other examples. One participant stated, “my diet is poor, so vitamins help make up the difference.” Regarding improved performance and increased energy, participants noted using dietary supplements to “feel better when performing high-intensity exercises,” “decrease perceived rate of exertion with caffeine,” and increase “muscular endurance during workouts” using creatine, among other examples. With the other rationales, participants reported using “electrolytes to help with hydration, muscle, and cardiovascular function due to the Florida climate,” “magnesium for sleep,” and “caffeine because I like coffee.” Participants also mentioned taking “multivitamins under the direction of a dermatologist to address some stress related hair loss,” and taking “vitamin B recommended by my [primary care provider].”

**Figure 5:**
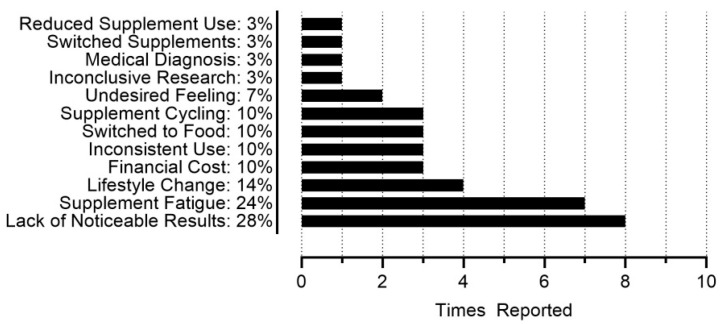
Frequencies for dietary supplement disuse rationale (n = 29).

Figure 5 provides the frequencies of final theme categorization from participants’ open responses regarding their rationale for dietary supplement disuse (n = 29). A lack of noticeable results, supplement fatigue (i.e., growing tired of taking supplements), and lifestyle change were the three most common reasons for participants discontinuing the use of at least one dietary supplement. Regarding a lack of noticeable results, participants reported becoming “lazy due to not seeing a short-term result,” having concerns that “green tea extract showed no noticeable benefit” and similarly never seeing or feeling a difference with branched chain amino acid (BCAA) supplementation. Regarding supplement fatigue, participants reported ceasing creatine supplementation “because I felt like I was already taking a lot” and discontinuing supplement use because “I got tired of them.” Lifestyle changes were exemplified by participants stating that they discontinued supplementation because they were “not competing at a high level anymore,” or because “weightlifting became less important to life,” among other examples. Participants also mentioned discontinuing supplements due to a “lack of money,” believing that they “get enough [BCAAs] from my diet already,” having “read that research was inconclusive if the doses you take in a BCAA supplement are actually enough to do what they say they do,” “just completely [forgetting] about them,” and “not [being] good at remembering to take them,” among other reasons.

## 4. Discussion

The purpose of this study was to detail the common dietary habits and body composition of HIFT athletes. Body composition varied widely, with a range of 6.5–37.6% body fat among all athletes. Dietary habits varied slightly, with a minority of participants regularly achieving the recommended two daily servings of fruits, vegetables, dairy, and whole grains. Dietary supplement use was common, for reasons like promoting health and wellness, improving recovery, and achieving dietary nutrient intake goals. Given the prevalent use of dietary supplements and their common rationale, many HIFT athletes may be relying on dietary supplements to address dietary inadequacies without changing dietary patterns.

Maxwell et al. [20] examined the sports nutrition knowledge, perceptions, and advice given by certified CrossFit^®^ trainers via an online survey, with a sample size of 289 participants. Using the Sports Nutrition Knowledge quiz, their participants scored 65.3 ± 12.4% correct yet gave a self-rated grade of a “B+” (~85–89% correct) on their nutritional knowledge [20], indicating both a modest perceived and observed gap in nutritional knowledge. CrossFit^®^ trainers commonly promoted the Paleo diet [20], which coincides with the observed dietary trends in the present study. The Paleo diet is characterized by low intakes of dairy, whole grains, legumes, and processed foods [21]. Similar trends were observed in our sample, with a minority of participants reporting regular consumption of ≥2 servings of dairy, whole grains, vegetables, and regular consumption of processed meats (Table 4). Likewise, the elevated consumption of meat in the Paleo diet was observed in the present study, with 71% of participants consuming >8 ounces (>227 g) of meat per day (Table 4). However, participants in the present study were not asked if they adhered to the Paleo diet or any other specific diet, so we cannot definitively conclude if the Paleo diet was prevalent in our sample. Furthermore, the Paleo diet does emphasize consumption of fruits and non-legume vegetables [21], yet those food items were also in the minority for regular consumption with our sample. Similar challenges with adhering to dietary recommendations have been observed in the overall adult U.S. population [22], so without a control group it is unclear if the observed dietary patterns in HIFT athletes would differ from non-HIFT athletes. Nonetheless, it is likely that a mix of dietary recommendations are followed by HIFT athletes, which may lead to intakes of certain food groups below recommended levels. Maxwell et al. [20] noted a significant positive relationship between Sports Nutrition Knowledge scores and self-reported hours spent on nutrition education, suggesting that implementing sports nutrition education into certifications for HIFT trainers and coaches may help improve overall sports nutrition knowledge and close the potential nutrient gaps in their athletes’ diets.

It is likely that participants mitigated gaps in their nutritional intake using dietary supplements, with 51% (n = 29) of participants citing health & wellness and 32% (n = 18) citing a desire to achieve certain nutrient intake goals as their rationale for using dietary supplements (Figure 4). Approximately 92% of all participants reported current or previous dietary supplement use, with an average use of ~6 different dietary supplements per participant, and the highest single-participant use being 16 different supplements. This parallels previous research, as a scoping review by Martinho et al. [23] reported the prevalence of dietary supplement use for CrossFit^®^ athletes ranging from 25 to 98%, with a mean of 72%. Martinho et al. [23] also reported creatine and protein as the most prevalent dietary supplements used by CrossFit^®^ athletes across several different studies [8,24,25,26,27,28,29,30]. In the present study, whey/casein protein and creatine were the top reported dietary supplements, with caffeine and electrolyte drinks closely behind as next most prevalent (Figure 3). Many commonly reported dietary supplements could be found in the diet, such as fish oils/omega 3s, vitamin D, and magnesium, although supplementation remains a viable strategy for achieving high recommended intakes, such as with protein [31]. HIFT athletes could potentially utilize fewer dietary supplements and address their common rationale for discontinuing dietary supplement use (Figure 5) if their nutritional needs were met via a “food-first approach.” While many studies have focused on the effects of nutritional interventions and dietary supplements on HIFT performance [32], to the best of our knowledge, no study has examined potential reductions in dietary supplement use with a nutritional intervention or nutritional education, highlighting room for future research. The typical HIFT athlete appears to be moderately knowledgeable regarding nutrition and reliant on several dietary supplements at any given time to support their health and performance, yet the typical HIFT athlete does appear to vary regarding their body composition.

Menargues-Ramirez et al. [11] evaluated body composition among 27 competitive Spanish CrossFit^®^ athletes, concluding that the average CrossFit^®^ athlete has low levels of fat mass and a small relative size. Broader inclusion criteria in the present study likely led to our differing conclusions, as we observed a wide range of leanness and relative size among competitive and non-competitive HIFT athletes (Table 3). Compared to standards from the American College of Sports Medicine, regardless of age, the observed body fat percentages for male (6.5–27.6%) and female (10.6–37.6%) HIFT athletes range from “very lean”/“excellent” to “poor”/“very poor” [33]. Likewise, relative body size (i.e., BMI) ranged from normal weight to obese in men and from underweight to obese in women. While Menargues-Ramirez et al. [11] recruited CrossFit^®^ athletes with extensive competitive experience, the present sample largely included non-competitive HIFT athletes, and the majority of competitive HIFT athletes only competed at the local level. It is likely that at higher levels of competition, a more consistent phenotype of HIFT athletes would be observed, favoring a lean and muscular physique. However, when examining the typical HIFT athlete regardless of competition status, body composition does not appear to be uniform.

This study is not without limitations, as our use of the Siri 3-compartment model limited our ability to assess other aspects of body composition such as muscle mass and bone mineral density. While the Siri 3-compartment model is a reliable criterion method for assessing body composition, future work should employ a 4- or 5-compartment criterion model to investigate more nuances within body composition. Some nuances in dietary habits were also not captured due to our use of the REAP-S. The REAP-S is a type of food frequency questionnaire designed for cross-sectional use and validated against other standard food frequency questionnaires [15], and our use of the REAP-S with HIFT athletes was novel. Nonetheless, further examination of macro- and micronutrient intake among HIFT athletes may help better elucidate potential nutrient gaps and if HIFT athletes rely on dietary supplements to reach recommended nutrient intake levels. Our qualitative approach provided valuable insight on the rationales for supplement use and disuse; however, these qualitative results should be interpreted cautiously. Our smaller sample size, due to low responses to recruitment efforts within the community, may have affected our ability to reach data saturation. While our recruitment efforts targeted individuals that were representative of the typical HIFT athlete, we were limited to a single geographical area, so future research should aim to replicate our work within a larger, multi-site sample. This study was strengthened by its mixed-methods design, as Maxwell et al. [20] was one of the only other studies to do so, and we were further strengthened by our moderate sample size compared to many other studies on HIFT athletes. Future research should continue to strive for larger and more diverse samples of HIFT athletes, while also incorporating qualitative measures to capture the experiences and beliefs of their participants as well as multi-site research designs to include HIFT athletes from more than one geographic region. With larger sample sizes, future studies may also be better able to statistically compare dietary habits between competitive levels to provide a more nuanced view of common practices among HIFT athletes.

## 5. Conclusions

HIFT athletes present with a wide range of body compositions, ranging from very lean to obese. Participation in HIFT appears to overlap with common best practices for sports nutrition, but there may be room for improvement in meeting fruit, vegetable, dairy, and whole-grain recommendations. Given the average usage of approximately six different dietary supplements per athlete, and the qualitative responses from participants, some HIFT athletes may be relying on dietary supplements to address nutrient gaps rather than whole foods. Future research should explore sport nutrition education interventions among HIFT athletes and potential downstream effects of education on dietary practices and body composition.

## Figures and Tables

**Table 1:** Participation rates for popular HIFT brands.

Brand	n of Global Participants
CrossFit^®^	5,000,000
Hyrox^®^	>90,000
F45^®^	300,000

Note. These participation rates are self-reported by each organization [1,3,4]. High-intensity functional training (HIFT) athletes also participate within other brands that do not list their participation rates on their website (e.g., DEKA^®^ [5]) or within non-branded gyms/communities, such as bootcamps.

**Table 2:** Participant demographics (N = 62).

	Mean ± SD or n (%)
Age (years)	36.3 ± 11.7 (18–64)
Sex	Female = 26 (41.9%) Male = 36 (58.1%)
Ethnicity	Hispanic = 15 (24.2%) Non-Hispanic = 47 (75.8%)
Race	Asian = 7 (11.3%) Black = 1 (1.6%) Hispanic/Latino = 10 (16.1%) White = 38 (61.3%) Middle Eastern = 2 (3.2%) Mixed Race = 4 (6.5%)

Note. SD = standard deviation. The minimum and maximum values for age (i.e., range) are provided in parentheses.

**Table 3:** Body composition of participants.

	All (N = 62)	Male (n = 36)	Female (n = 26)
Body Mass (kg)	76.8 ± 15.5 (44.4–113.0)	86.2 ± 11.3 (65.9–113.0)	63.8 ± 10.1 (44.4–94.3)
Body Mass Index (kg/m^2^)	25.8 ± 3.4 (18.2–32.7)	27.2 ± 2.7 (22.2–32.7)	23.9 ± 3.4 (18.2–32.2)
Body Fat %	20.4 ± 6.9 (6.5–37.6)	17.7 ± 5.6 (6.5–27.6)	24.3 ± 6.9 (10.6–37.6)
Fat Mass (kg)	15.6 ± 5.9 (5.0–29.9)	15.5 ± 5.9 (5.0–28.7)	15.8 ± 6.2 (5.1–29.9)
Fat-Free Mass (kg)	61.2 ± 13.7 (36.8–94.8)	70.8 ± 8.3 (55.8–94.8)	48.0 ± 6.5 (36.8–64.4)
Total Body Density (kg/L)	1.05 ± 0.02 (1.02–1.09)	1.06 ± 0.01 (1.03–1.09)	1.04 ± 0.02 (1.02–1.08)
Total Body Volume (L)	73.0 ± 14.6 (41.5–106.0)	81.5 ± 10.9 (61.9–106.0)	61.1 ± 10.2 (41.5–91.9)
Total Body Water (L)	44.6 ± 9.9 (27.3–68.2)	51.6 ± 6.1 (40.7–68.2)	35.1 ± 4.8 (27.3–47.6)

Note. Data are presented as mean ± standard deviation, with minimum and maximum values in parentheses (i.e., range). Total body volume and total body density were assessed via BodPod. Total body water was assessed via bioelectrical impedance analysis. All other body composition parameters were derived from Siri 3-Compartment model.

**Table 4:** Participant typical dietary habits from the REAP-S (N = 62).

	Usually/Often	Sometimes	Rarely/Never
Skip breakfast?	9 (14.5%)	12 (19.4%)	41 (66.1%)
Eat 4 or more meals from sit-down or take out restaurants?	3 (4.8%)	16 (25.8%)	43 (69.4%)
Eat less than 2 servings of whole-grain products or high-fiber starches a day?	12 (19.4%)	24 (38.7%)	26 (41.9%)
Eat less than 2 servings of fruit a day?	15 (24.2%)	25 (40.3%)	22 (35.5%)
Eat less than 2 servings of vegetables a day?	10 (16.1%)	22 (35.5%)	30 (48.4%)
Eat or drink less than 2 servings of milk, yogurt, or cheese a day?	14 (22.6%)	18 (29.0%)	30 (48.4%)
Eat more than 8 ounces (~227 g) of meat, chicken, turkey or fish per day?	44 (71.0%)	15 (24.2%)	3 (4.8%)
Use regular processed meats (e.g., bologna, salami, hotdogs, or bacon) instead of low-fat processed meats (like roast beef, turkey, lean ham)?	3 (4.8%)	15 (24.2%)	44 (71.0%)
Eat fried foods?	6 (9.7%)	25 (40.3%)	31 (50.0%)
Eat regular snack chips, popcorn, or nuts instead of pretzels, low-fat chips or low-fat crackers, air-popped popcorn?	4 (6.5%)	23 (37.1%)	35 (56.4%)
Add butter, margarine or oil to bread, potatoes, rice or vegetables at the table?	7 (11.3%)	23 (37.1%)	32 (51.6%)
Eat sweets like cake, cookies, pastries, or candies more than 2 times per day.	5 (8.1%)	23 (37.1%)	34 (54.8%)
Drink 16 (~473 mL) ounces or more of non-diet soda or fruit drink/punch per day?	1 (1.6%)	6 (9.7%)	55 (88.7%)

Note. Data are presented as n (%). REAP-S = Rapid Eating Assessment for Participants, Shortened Version.

## Data Availability

The data presented in this study are available on request from the corresponding author (D.H.F.). The data are not publicly available due to ethical reasons.

## Abbreviations

The following abbreviations are used in this manuscript:

BMI	Body Mass Index
HIFT	High-Intensity Functional Training
SNK	Sports Nutrition Knowledge

## Appendix A

**Table A1:** Body composition estimates from the InBody 970^®^.

	All (N = 62)	Male (n = 36)	Female (n = 26)
Body Mass (kg)	76.9 ± 15.5 (44.4–113.0)	86.3 ± 11.1 (65.9–113.0)	63.8 ± 10.1 (44.4–94.3)
Body Fat Percentage (%)	20.8 ± 7.1 (6.0–39.8)	18.1 ± 5.6 (6.0–28.5)	24.4 ± 7.3 (11.0–39.8)
Fat Mass (kg)	15.9 ± 6.1 (4.5–29.8)	15.9 ± 6.0 (4.5–29.8)	15.9 ± 6.4 (4.9–29.7)
Fat-Free Mass (kg)	61.0 ± 13.6 (37.3–92.9)	70.4 ± 8.4 (55.5–92.9)	47.9 ± 6.6 (37.3–65.0)
Impedance (Ω)	544.0 ± 80.6 (387.0–764.0)	494.0 ± 52.8 (387.0–607.0)	611.0 ± 60.0 (510.0–764.0)
Resistance (Ω)	540.0 ± 80.5 (383.0–761.0)	490.0 ± 52.6 (383.0–603.0)	608.0 ± 59.9 (507.0–761)
Reactance (Ω)	61.6 ± 7.5 (41.8–76.0)	59.5 ± 6.8 (41.8–72.5)	64.4 ± 7.6 (46.7–76.0)
Impedance Ratio	0.768 ± 0.025 (0.716–0.840)	0.755 ± 0.019 (0.716–0.797)	0.785 ± 0.021 (0.753–0.840)
Phase Angle (°)	6.58 ± 0.75 (4.50–8.20)	6.94 ± 0.60 (5.60–8.20)	6.08 ± 0.64 (4.50–7.00)

Note. Data are presented as mean ± standard deviation, with minimum and maximum values in parentheses (i.e., range). Impedance, resistance, and reactance were extracted at 50 kHz. Impedance ratio was calculated as Z_250kHz_/Z_5kHz_. For impedance, resistance, reactance, and impedance ratio in the “All” and “Male” categories, sample sizes were N = 61 and n = 35, respectively, due to missing data from data collection.

## References

[1] What is CrossFit?. https://www.crossfit.com/what-is-crossfit.

[2] Mangine G.T., Stratton M.T., Almeda C.G., Roberts M.D., Esmat T.A., VanDusseldorp T.A., Feito Y. (2020). Physiological differences between advanced CrossFit athletes, recreational CrossFit participants, and physically-active adults. PLoS ONE.

[3] HYROX: The History. https://hyrox.com/the-history/.

[4] F45: International Franchise Expo. https://f45training.com/ife/.

[5] DEKA. https://www.spartan.com/en/deka.

[6] de Souza R.A.S., da Silva A.G., de Souza M.F., Souza L.K.F., Roschel H., da Silva S.F., Saunders B. (2021). A Systematic Review of CrossFit(R) Workouts and Dietary and Supplementation Interventions to Guide Nutritional Strategies and Future Research in CrossFit(R). Int. J. Sport. Nutr. Exerc. Metab..

[7] Knapik J.J., Steelman R.A., Hoedebecke S.S., Austin K.G., Farina E.K., Lieberman H.R. (2016). Prevalence of Dietary Supplement Use by Athletes: Systematic Review and Meta-Analysis. Sports Med..

[8] Brisebois M., Kramer S., Lindsay K.G., Wu C.-T., Kamla J. (2022). Dietary practices and supplement use among CrossFit® participants. J. Int. Soc. Sports Nutr..

[9] Gogojewicz A., Śliwicka E., Durkalec-Michalski K. (2020). Assessment of dietary intake and nutritional status in CrossFit-trained individuals: A descriptive study. Int. J. Environ. Res. Public Health.

[10] Pearson R.C., Jenkins N.T. (2022). Dietary Intake of Adults Who Participate in CrossFit^®^ Exercise Regimens. Sports.

[11] Menargues-Ramírez R., Sospedra I., Holway F., Hurtado-Sánchez J.A., Martínez-Sanz J.M. (2022). Evaluation of body composition in CrossFit^®^ athletes and the relation with their results in official training. Int. J. Environ. Res. Public Health.

[12] de Sousa A.F., dos Santos G.B., dos Reis T., Valerino A.J., Del Rosso S., Boullosa D.A. (2016). Differences in Physical Fitness between Recreational CrossFit^®^ and Resistance Trained Individuals. J. Exerc. Physiol. Online.

[13] Cebrián-Ponce Á., Serafini S., Petri C., Carrasco-Marginet M., Izzicupo P., Mascherini G. (2024). Somatotype and bioelectrical impedance vector analysis of Italian CrossFit^®^ practitioners. Heliyon.

[14] Lafontant K. (2025). Created in BioRender. Flow of Study Procedures. https://BioRender.com/uuhihve.

[15] Segal-Isaacson C., Wylie-Rosett J., Gans K.M. (2004). Validation of a short dietary assessment questionnaire: The Rapid Eating and Activity Assessment for Participants short version (REAP-S). Diabetes Educ..

[16] Lukaski H.C., Kyle U.G., Kondrup J. (2017). Assessment of adult malnutrition and prognosis with bioelectrical impedance analysis: Phase angle and impedance ratio. Curr. Opin. Clin. Nutr. Metab. Care.

[17] Lukaski H.C., Garcia-Almeida J.M. (2023). Phase angle in applications of bioimpedance in health and disease. Rev. Endocr. Metab. Disord..

[18] Siri W., Brozek J., Henschel A. (1961). Techniques for Measuring Body Composition.

[19] Love J., Droppmann D., Selker R., Gallucci M., Jentschke S., Balci S., Seol H., Agosti M. (2024). Jamovi®.

[20] Maxwell C., Ruth K., Friesen C. (2017). Sports Nutrition Knowledge, Perceptions, Resources, and Advice Given by Certified CrossFit Trainers. Sports.

[21] Singh A., Singh D. (2023). The Paleolithic Diet. Cureus.

[22] King D.E., Mainous A.G., Carnemolla M., Everett C.J. (2009). Adherence to healthy lifestyle habits in US adults, 1988–2006. Am. J. Med..

[23] Martinho D.V., Rebelo A., Clemente F.M., Costa R., Gouveia E.R., Field A., Casonatto J., van den Hoek D., Durkalec-Michalsk K., Ormsbee M.J. (2025). Nutrition in CrossFit(R)-scientific evidence and practical perspectives: A systematic scoping review. J. Int. Soc. Sports Nutr..

[24] Freitas J.C.R.d.S.O. (2016). Potencial Ergogénico e Uso da Creatinina e da Beta-Alanina no Contexto do CrossFit e da Musculação. https://core.ac.uk/download/pdf/148829073.pdf.

[25] de Lima Lins T.C., de Souza L.P.V. (2019). Dieta pré e pós treino em praticantes de crossfit^®^: Um perfil qualitativo do consumo de alimentos e suplementos. RBNE-Rev. Bras. Nutr. Esportiva.

[26] Fayad D. (2019). A Influência da Estratégia Nutricional no Rendimento de Atletas Competitivos de Crossfit. https://repositorio.uniceub.br/jspui/handle/prefix/13504.

[27] Brescansin B.M., Naziazeno R.F.T., de Miranda T.V. (2019). Análise do perfil alimentar de praticantes de CrossFit na região de Belém do Pará. RBNE-Rev. Bras. Nutr. Esportiva.

[28] Rebouças Filho M., Mendonça L.P. (2022). Perfil alimentar e utilização de suplementos nutricionais de praticantes de cross training do Box Crossfit Sertão no município de Mossoró. RBNE-Rev. Bras. Nutr. Esportiva.

[29] Comerlatto V., Zanella P.B., Hoefel A.L. (2023). Crossfit^®^ practitioners profile with regard to the prevalence of use of dietary supplements and anabolic androgenic steroids as ergogenic resources. RBNE-Rev. Bras. Nutr. Esportiva.

[30] Higino D.D., Freitas R.F. (2021). Prevalência e fatores associados ao uso de suplementos alimentares e esteroides anabólicos androgênicos em praticantes de CrossFIT. RBNE-Rev. Bras. Nutr. Esportiva.

[31] Jager R., Kerksick C.M., Campbell B.I., Cribb P.J., Wells S.D., Skwiat T.M., Purpura M., Ziegenfuss T.N., Ferrando A.A., Arent S.M. (2017). International Society of Sports Nutrition Position Stand: Protein and exercise. J. Int. Soc. Sports Nutr..

[32] dos Santos Quaresma M.V., Marques C.G., Nakamoto F.P. (2021). Effects of diet interventions, dietary supplements, and performance-enhancing substances on the performance of CrossFit-trained individuals: A systematic review of clinical studies. Nutrition.

[33] Liguori G., American College of Sports Medicine (2021). ACSM’s Guidelines for Exercise Testing and Prescription.

